# Interaction between Red Meat Intake and *NAT2* Genotype in Increasing the Risk of Colorectal Cancer in Japanese and African Americans

**DOI:** 10.1371/journal.pone.0144955

**Published:** 2015-12-18

**Authors:** Hansong Wang, Motoki Iwasaki, Christopher A. Haiman, Suminori Kono, Lynne R. Wilkens, Temitope O. Keku, Sonja I. Berndt, Shoichiro Tsugane, Loïc Le Marchand

**Affiliations:** 1 Epidemiology Program, University of Hawaii Cancer Center, Honolulu, Hawaii, United States of America; 2 Research Center for Cancer Prevention and Screening, National Cancer Center, Tokyo, Japan; 3 Department of Preventive Medicine, Keck School of Medicine and Norris Comprehensive Cancer Center, University of Southern California, Los Angeles, California, United States of America; 4 Department of Preventive Medicine, Graduate School of Medical Sciences, Kyushu University, Fukuoka, Japan; 5 Center for Gastrointestinal Biology and Disease, University of North Carolina, Chapel Hill, North Carolina, United States of America; 6 Division of Cancer Epidemiology and Genetics, National Cancer Institute, National Institutes of Health, Bethesda, Maryland, United States of America; University of Aberdeen, UNITED KINGDOM

## Abstract

Heterocyclic aromatic amines formed in cooked meat may be an underlying mechanism for the red meat-colorectal cancer (CRC) association. These compounds require bioactivaction by *N*-acetyltransferase 2 (NAT2). An interaction effect between red meat consumption and NAT2 in increasing CRC risk has been inconsistently reported in whites. We investigated this interaction in two populations in which the high-activity rapid NAT2 phenotype is 10- and 2-fold more common than in whites. We meta-analyzed four studies of Japanese (2,217 cases, 3,788 controls) and three studies of African Americans (527 cases, 4,527 controls). NAT2 phenotype was inferred from an optimized seven-SNP genotyping panel. Processed and total red meat intakes were associated with an increased CRC risk in Japanese and in both ethnic groups combined (P’s ≤ 0.002). We observed an interaction between processed meat intake and NAT2 in Japanese (P = 0.04), African Americans (P = 0.02), and in both groups combined (P = 0.006). The association of processed meat with CRC was strongest among individuals with the rapid NAT2 phenotype (combined analysis, OR for highest vs. lowest quartile: 1.62, 95% CI: 1.28–2.05; P_trend_ = 8.0×10^−5^), intermediate among those with the intermediate NAT2 phenotype (1.29, 95% CI: 1.05–1.59; P_trend_ = 0.05) and null among those with the slow phenotype (P_trend_ = 0.45). A similar interaction was found for NAT2 and total red meat (P_interaction_ = 0.03). Our findings support a role for NAT2 in modifying the association between red meat consumption and CRC in Japanese and African Americans.

## Introduction

Colorectal cancer (CRC) is a leading cause of cancer mortality worldwide, with approximately 1.4 million new cases and 694,000 disease-specific deaths in 2012[1]. In non-western countries and particularly in Asia, as more people adopt a high-energy diet and low physical activity that are part of the “western” lifestyle, the incidence of CRC has been increasing, with the highest incidence rates currently being reported from Japan. Red meat and processed meat consumption are established risk factors for CRC [2–5]. One hypothesized mechanism for this association is through exposure to heterocyclic aromatic amines (HAAs), which are formed when meat is cooked at high temperature for a long duration [6–8].

*N*-acetyltransferase 2 (NAT2) has been shown to play a critical role in the bioactivation of HAAs. *N*-hydroxylated HAA metabolites are substrates for *O*-acetylation primarily by NAT2 to form the reactive *N*-acetoxy species which bind to DNA. *N*-acetylation activity can be determined by dosing subjects with a substrate, such as isoniazid, sulfamethazine or caffeine, and measuring urinary metabolites [9]. More than 25 genetic polymorphisms have been identified for *NAT2* that can affect the catalytic activity of NATs toward HAAs. Several genotyping panels have been used to classify *NAT2* genotypes and infer phenotype. A panel of seven SNPs for *NAT2* have been shown to be optimal [10,11], although a single SNP (rs1495741) has been suggested to be adequate for *NAT2* in Europeans [12].

The frequency of the slow NAT2 phenotype varies markedly across populations, from approximately 5% in Canadian Eskimos, to 10% in Japanese, 50% in Europeans and 90% in North Africans [13]. It is notable that populations with the highest frequencies for the rapid acetylation phenotype also have the highest CRC incidence rates in the world (Native Alaskans and Japanese Americans), and those with the lowest rapid acetylation phenotype frequencies have very low CRC rates (e.g., in North Africa)[1,14]. Moreover, the raising trends in colon cancer incidence and mortality in Japan have closely paralleled the increase in red meat intake with a 20 year lag [15]. An ecological study showed that NAT2 phenotype significantly improved the international correlation that exists between country-specific meat consumption and CRC incidence [16]. A number of studies have suggested a stronger association between CRC or its precursor, adenoma, and red meat consumption among individuals with the rapid NAT2 phenotype [17–22], although not consistently [23–26]. This lack of consistency could be due to multiple aspects of study heterogeneity, such as differences in inferring NAT2 activity from genotype, study population, sample size, and in analysis strategies (e.g. grouping the intermediate and rapid phenotypes for Europeans, a population with a low frequency for the rapid phenotype).

We report on the modifying effect of NAT2 on the association of red meat intake on the risk of colorectal cancer in a genome-wide association study (GWAS) conducted in Japanese and African Americans. These two populations have high rates of CRC and a frequency of rapid acetylator phenotype which is 10- and 2-fold greater than in whites, respectively.

## Results

The Japanese samples included 2,217 CRC cases and 3,788 controls from the Multiethnic Cohort study (MEC), the Fukuoka Colorectal Cancer Study (Fukuoka), the Japan Public Health Center cohort study (JPHC) and the Nagano Colorectal Cancer Study (Nagano). The African American samples included 527 cases and 4,527 controls from the MEC, the University of North Carolina Rectal Cancer Study (UNC) and the Prostate, Lung, Colorectal and Ovarian Cancer Screening (PLCO) Trial. Characteristics of study participants are shown by ethnic/racial group and study in Table 1. The frequency of the rapid NAT2 phenotype varied across studies from 46.4% to 48.4% in Japanese and from 6.5% to 10.7% in African Americans, consistent with previous studies [27]. NAT2 levels were not significantly associated with CRC in each population or when the two were combined (P’s > 0.19, S1 Table).

**Table 1:** Characteristics of study subjects.

	Japanese				African American		
	Fukuoka	Nagano	JPHC	MEC	MEC	PLCO	UNC
No. of Cases	662	105	653	797	342	76	109
No. of Controls	749	102	640	2297	4328	94	105
Total	1411	207	1293	3094	4670	170	214
Female (%)	37.0	35.7	48.1	45.2	34.4	52.9	45.8
Age (year)	59.5 (10)	59.4 (8.8)	61.2 (9.2)	69.6 (8.5)	68.9 (8.2)	65.5 (5.5)	62.5 (10.1)
BMI (kg/m2)	23.2 (3.1)	23.2 (2.9)	23.8 (3)	24.7 (3.7)	28.1 (4.9)	29.7 (6.4)	30.1 (6.7)
Ever smoker (%)	59.3	49.8	39.6	52.5	66.9	62.4	58.4
Regular aspirin use (%)	5	na	na	38	62	56	72
Processed meat intake (g/day)	8.0 (9.5)	7.0 (9.6)	3.2 (3.5)	16.6 (15.0)	20.6 (22.1)	24.6 (22.9)	29.6 (52.5)
Red meat without processed meat (g/day)	43.0 (30.1)	45.4 (43.1)	15.3 (11.8)	37.2 (27.2)	40.0 (37.5)	49.4 (43.4)	52.5 (45.1)
Total red meat intake (g/day)	51.0 (34.4)	52.3 (46.8)	18.5 (13.3)	53.8 (38.1)	60.6 (52.4)	74.1 (58.2)	82.2 (78.1)
Folate from foods (mcg/day)	399 (146)	449 (293)	307 (117)	337 (186)	373 (243)	351 (169)	na
Folate from foods and supplement (mcg/day)(DFE)	na	na	na	608 (471)	606 (496)	529 (348)	606 (277)
Calcium from foods (mg/day)	693 (272)	606 (396)	440 (257)	637 (306)	732 (436)	799 (453)	780 (384)
Calcium from foods and supplement (mg/day)	na	na	na	973 (632)	906 (550)	924 (566)	870 (455)
Dietary fiber (g/day)	15.3 (5.8)	14.6 (11.9)	7.7 (3.1)	22.2 (11.3)	24.5 (14.9)	22.1 (11.6)	20.8 (10.0)
NAT2 Phenotype							
Slow (%)	10.3	8.2	9.7	10.1	15.7	15.3	14.0
Intermediate (%)	43.3	45.9	41.9	43.4	73.6	78.2	77.1
Rapid (%)	46.4	45.9	48.4	46.4	10.7	6.5	8.9

Abbreviations: Fukuoka: the Fukuoka Colorectal Cancer Study; Nagano: the Nagano Colorectal Cancer Study; JPHC: Japan Public Health Center-Based prospective study; MEC: Multiethnic Cohort Study; PLCO: Prostate, Lung, Colorectal, and Ovarian Cancer Screening Trial; UNC: The North Carolina Rectal Cancer Study.

Values are means (standard deviations) unless specified otherwise.

The study-specific cut-points for creating the 4-level meat variables are shown in S2 Table. Greater intakes of processed meat, red meat without processed meat and total red meat were all statistically significantly associated with CRC risk in Japanese and when the Japanese were combined with African Americans (P_trend_ ≤ 0.0051) (Table 2). In African Americans alone, the associations were of the same direction but with P-values > 0.05, partly reflecting the relatively small number of cases in this group. The odds ratio (OR) for each quartile increase in processed meat level was 1.10 [95% confidence interval (CI): 1.05, 1.16] in Japanese (P_trend_ = 0.0002), 1.02 (95% CI: 0.93, 1.13) in African Americans (P_trend_ = 0.62) and 1.09 (95% CI: 1.04, 1.14) in the combined analysis (P_trend_ = 0.0004). The association for red meat without processed meat was similar but of somewhat lesser magnitude (Table 2). No within-ethnic group heterogeneity across studies was observed (P_het_’s > 0.39). Similarly, no heterogeneity was detected by study design (prospective vs. case-control studies) (P_het_’s>0.24). There was weak evidence for between-ethnic group heterogeneity (I^2^ = 44%) for the association between processed meat and CRC, but not for the other two meat variables. Adjusting for additional risk factors in these data did not materially change the results (S3 Table), except that the between-ethnic group heterogeneity was weakened (I^2^ = 8%) for the association between processed meat and CRC.

**Table 2:** Association (odds ratios and 95% confidence interval) between meat intakes and colorectal cancer.

	Cases	Controls	Quartile 1	Quartile 2	Quartile 3	Quartile 4	P_trend_	I^2^ (%)
**Processed meat**								
Japanese	2186	3736	1.0	1.02 (0.88, 1.19)	1.11 (0.95, 1.30)	1.38 (1.17, 1.62)	0.0002	
African American	466	4356	1.0	1.26 (0.94, 1.69)	1.03 (0.76, 1.39)	1.16 (0.85, 1.57)	0.62	
Combined	2652	8092	1.0	1.07 (0.93, 1.23)	1.09 (0.95, 1.25)	1.32 (1.14, 1.53)	0.0004	44
**Red meat without processed meat**								
Japanese	2186	3736	1.0	1.12 (0.96, 1.32)	1.14 (0.97, 1.33)	1.27 (1.08, 1.49)	0.006	
African American	466	4356	1.0	1.34 (1.00, 1.82)	1.21 (0.9, 1.64)	1.18 (0.87, 1.59)	0.40	
Combined	2652	8092	1.0	1.17 (1.02, 1.34)	1.15 (1.00, 1.33)	1.24 (1.08, 1.44)	0.0051	0
**Total red meat**								
Japanese	2186	3736	1.0	1.18 (1.00, 1.38)	1.11 (0.95, 1.30)	1.33 (1.13, 1.57)	0.002	
African American	466	4356	1.0	1.59 (1.18, 2.14)	1.31 (0.97, 1.76)	1.28 (0.95, 1.73)	0.21	
Combined	2652	8092	1.0	1.26 (1.09, 1.45)	1.15 (1.00, 1.32)	1.32 (1.15, 1.52)	0.001	0

Adjusted for age, sex, BMI (continuous), the first 4 principal components and study separately in the Japanese and AA.

Sample sizes were reduced due to missing values in meat intakes.

The risk estimates for these associations also did not materially change in analyses restricted to colon cancer or rectal cancer for the Japanese studies, which were large enough to allow for such subsite-specific analyses. For example, the OR for processed meat was 1.09 (95% CI: 1.03, 1.16, P = 0.003) for colon cancer and 1.14 (95% CI: 1.06, 1.23, P = 0.001) for rectal cancer.

We observed a statistically significant interaction between processed meat and NAT2 phenotype on the risk of CRC in Japanese (P_interaction_ = 0.044), in African Americans (P_interaction_ = 0.018) and in both ethnic groups combined (P_interaction_ = 0.006) (Table 3). In the Japanese, the effect of processed meat was strongest in subjects with the rapid NAT2 phenotype (OR for the highest to lowest quartile: 1.61, 95% CI: 1.26, 2.06; P_trend_ = 1.8×10^−4^). The corresponding OR for Japanese with the slow NAT2 phenotype was 1.06 (95% CI: 0.63, 1.79); P_trend_ = 0.70). In African Americans, for whom the number of cases was much smaller, processed meat was not significantly associated with CRC in any NAT2 subgroup (all P_trend_ > 0.05). Nonetheless, an interaction effect was observed between quartile of processed meat and NAT2 level (P_interaction_ = 0.018). Results in the analysis combining both Japanese and African Americans for processed red meat resembled those in the Japanese with weak between-ethnic group heterogeneity (I^2^ = 49.7%). The interaction between total red meat and NAT2 was also significant when both ethnic groups were combined (P_interaction_ = 0.03). Total red meat intake was associated with CRC risk in both the rapid and intermediate NAT2 categories (P_trend_ = 0.003 and 0.015, respectively) but not in the slow NAT2 category (P_trend_ = 0.53). Since we did not find a statistically significant interaction between NAT2 and red meat without processed meat (P_interaction_’s > 0.17), the interaction between total red meat and NAT2 on CRC risk in the combined analysis was mostly reflective of that between processed meat and NAT2. The interactions between NAT2 and red meat did not differ by study design (prospective vs case-control) (P_het_’s > 0.47).

**Table 3:** Association of meat intake with colorectal cancer, stratified by NAT2 phenotypes.

	Group	NAT2	Cases	Controls	Quartile 1	Quartile 2	Quartile 3	Quartile 4	P_trend_
**Processed meat**	**Japanese**	S	213	380	1.0	0.89 (0.55, 1.44)	1.07 (0.66, 1.72)	1.06 (0.63, 1.79)	0.70
	(P_interaction_ = 0.044)	I	944	1613	1.0	0.91 (0.72, 1.14)	0.97 (0.77, 1.23)	1.27 (0.98, 1.64)	0.10
		R	1029	1743	1.0	1.19 (0.95, 1.50)	1.26 (1.01, 1.59)	1.61 (1.26, 2.06)	0.00018
	**African American**	S	82	677	1.0	0.77 (0.38, 1.57)	0.68 (0.34, 1.35)	0.44 (0.19, 1.01)	0.051
	(P_interaction_ = 0.018)	I	344	3216	1.0	1.46 (1.03, 2.05)	1.07 (0.75, 1.52)	1.34 (0.94, 1.91)	0.32
		R	40	463	1.0	0.99 (0.32, 3.01)	1.83 (0.64, 5.17)	1.78 (0.62, 5.13)	0.18
	**Combined**	S	295	1057	1.0	0.85 (0.57, 1.26)	0.92 (0.62, 1.36)	0.83 (0.53, 1.29)	0.453
	(P_interaction_ = 0.006, I^2^ = 49.7%)	I	1288	4829	1.0	1.05 (0.87, 1.28)	1.00 (0.82, 1.22)	1.29 (1.05, 1.59)	0.053
		R	1069	2206	1.0	1.18 (0.95, 1.48)	1.28 (1.03, 1.6)	1.62 (1.28, 2.05)	8x10^−5^
**Red meat without processed meat**	**Japanese**	S	213	380	1.0	1.01 (0.62, 1.65)	1.11 (0.66, 1.86)	1.08 (0.65, 1.79)	0.71
	(P_interaction_ = 0.22)	I	944	1613	1.0	1.02 (0.80, 1.30)	1.15 (0.90, 1.46)	1.20 (0.93, 1.54)	0.10
		R	1029	1743	1.0	1.27 (1.00, 1.60)	1.15 (0.91, 1.46)	1.41 (1.11, 1.80)	0.014
	**African American**	S	82	677	1.0	0.60 (0.27, 1.31)	0.76 (0.38, 1.52)	0.65 (0.31, 1.33)	0.32
	(P_interaction_ = 0.55)	I	344	3216	1.0	1.72 (1.20, 2.44)	1.43 (1.00, 2.03)	1.43 (1.00, 2.05)	0.12
		R	40	463	1.0	0.91 (0.35, 2.38)	0.87 (0.32, 2.36)	0.79 (0.30, 2.12)	0.64
	**Combined**	S	295	1057	1.0	0.87 (0.58, 1.32)	0.97 (0.64, 1.46)	0.91 (0.60, 1.38)	0.789
	(P_interaction_ = 0.17, I^2^ = 0)	I	1288	4829	1.0	1.20 (0.98, 1.47)	1.23 (1.01, 1.50)	1.27 (1.04, 1.56)	0.026
		R	1069	2206	1.0	1.24 (0.99, 1.56)	1.14 (0.91, 1.43)	1.37 (1.09, 1.73)	0.023
**Total red meat**	**Japanese**	S	213	380	1.0	1.56 (0.95, 2.56)	1.09 (0.65, 1.85)	1.19 (0.72, 1.95)	0.79
	(P_interaction_ = 0.075)	I	944	1613	1.0	1.12 (0.88, 1.43)	1.06 (0.83, 1.34)	1.25 (0.97, 1.61)	0.14
		R	1029	1743	1.0	1.16 (0.92, 1.46)	1.18 (0.93, 1.48)	1.48 (1.17, 1.88)	0.0021
	**African American**	S	82	677	1.0	0.79 (0.37, 1.70)	0.96 (0.48, 1.89)	0.50 (0.23, 1.08)	0.14
	(P_interaction_ = 0.17)	I	344	3216	1.0	1.78 (1.25, 2.53)	1.47 (1.03, 2.10)	1.56 (1.09, 2.21)	0.04
		R	40	463	1.0	2.38 (0.91, 6.21)	0.78 (0.24, 2.50)	1.39 (0.51, 3.80)	0.97
	**Combined**	S	295	1057	1.0	1.28 (0.84, 1.93)	1.04 (0.69, 1.58)	0.92 (0.60, 1.40)	0.534
	(P_interaction_ = 0.030, I^2^ = 0)	I	1288	4829	1.0	1.30 (1.06, 1.59)	1.17 (0.96, 1.43)	1.35 (1.10, 1.65)	0.015
		R	1069	2206	1.0	1.21 (0.96, 1.51)	1.16 (0.92, 1.45)	1.47 (1.17, 1.86)	0.003

S: Slow; I: Intermediate; R: Rapid

Adjusted for age, sex, BMI (continuous), the first 4 principal components and study separately in the Japanese and African Americans.

P_interaction_ was from a Wald test and was verified by a likelihood ratio test within each ethnic group.

In a sensitivity analysis, the OR estimates for the meat variables within each NAT2 category did not change materially (<12%) after adjustment for additional CRC risk factors in the Japanese, although the p-values for the interactions between NAT2 and processed meat changed from 0.044 to 0.075 (S4 Table). In subsite analyses in the Japanese, the interactions between processed meat and NAT2 was statistically significant for colon cancer (P = 0.04) but not for rectal cancer (P = 0.41), although the ORs for processed meat within NAT2 category in each subsite analysis were similar to those for colorectal cancer (changes <9%).

It was reported that in Caucasians, rs1495741 predicts the NAT2 phenotype inferred from the 7-SNP panel well, with sensitivity and specificity for using the genotype AA to assign the NAT2 slow phenotype of 99% and 95%, respectively [12]. Similarly in our data, high prediction accuracy was observed in Japanese but not in African Americans (S5 Table). The corresponding sensitivity and specificity was 96.8% and 98.9% in Japanese and 97.1% and 70.5% in African Americans. The agreement rate, i.e. the proportion of subjects with the AA, AG or GG genotype who are inferred to the same slow, intermediate or rapid NAT2 phenotype category by both methods, was 95.8% in Japanese and 67.7% in African Americans.

## Discussion

In this large study of Japanese and African Americans, two populations at high risk for CRC and with a high and intermediate frequency of NAT2 rapid phenotype, respectively, we observed a statistically significant interaction between processed meat intake and NAT2 activity in each population, and with total red meat intake in both populations combined. The interactions for processed meat and total red meat appeared to be dose-dependent since their associations with CRC were strongest among individuals with the rapid NAT2 phenotype, intermediate among individuals with the intermediate NAT2 phenotype, and non-significant among those with the slow NAT2 phenotype.

*N*-acetyltransferases (NATs) are thought to play a critical role in the genotoxicity of HAAs. *N*-hydroxylated HAA metabolites are substrates for *O*-acetylation primarily by NAT2 to form the reactive *N*-acetoxy species which bind to DNA. As a result, cancer risk may be particularly elevated in individuals who are rapid acetylators. A number of case-control and prospective studies have suggested an increased risk of colorectal cancer for individuals with the rapid acetylator status, assessed by phenotyping or genotyping. However, meta-analyses of the literature on NAT2 acetylator status (considered as rapid/intermediate vs. slow genotype or phenotype) have typically not confirmed this association [28–30]. Our study was also consistent with a lack of main effect for NAT2 on CRC risk.

Consistent with our results, interactions were suggested in a number of previous studies between intake of red meat, well-done meat or HAA and NAT2 acetylator status on the risk of colorectal neoplasia [17–22]. However, other studies, some with large sample sizes, failed to replicate this interaction between meat intake and NAT2 on colorectal cancer or adenoma risk [23–26].

Unfortunately, adding to the difficulty in interpreting past data, only a few studies and no meta-analysis or pooled analyses have reported risk estimates specifically for rapid acetylators (homozygous for the NAT2*4 allele) (the subset expected to be at the greatest risk), as grouping intermediate with rapid acetylators has been the norm, probably because most past studies were conducted in whites, a population with a low frequency of rapid NAT2 phenotype. A power computation suggests that the replication (with 80% power) of an interaction effect of the magnitude observed among Japanese in our study in a European-descent population would require about 6,000 cases and as many controls. The largest analysis to date [26], which combined 9 studies restricted to populations of European ancestry to examine the interaction between red meat intake and NAT2 on the risk of CRC exceeded this sample size (8,290 cases and 9,115 controls) and did not detect any significant interaction. It is possible that the modifying effect of NAT2 on the association between red meat intake and CRC is population-specific due to differences in cooking practices and, thus, HAA intake across populations and/or other modifying factors. On-going efforts to develop biomarkers of long term exposure to HAAs may be useful in clarifying these population differences [31].

In addition to its large sample size and the ability to distinguish the three NAT2 phenotypes in our analysis, the current study presents a number of strengths. We were able to harmonize exposure variables collected from various populations and differing study instruments, which allows for more robust and generalizable findings. We were also able to consider multiple risk factors for CRC as potential confounders, ensuring the independence of the observed effects.

Some study limitations deserve consideration. There was variation in the comprehensiveness of the dietary data used in this analysis which required that we used study-specific quantiles because of differences in methodology across studies. This is exemplified in JPHC for which we used the baseline data, which were collected using a ~50 items food-frequency questionnaire. We note that the follow-up survey in JPHC used a more detailed questionnaire (~150 items), similar to the one used in the Nagano Study, and yielded similar intake values as in the Nagano study [32], confirming that the intake variation in our data was most likely due to instrument differences and not to selection bias. Moreover, not all studies included here attempted to quantify HAA intake based on meat cooking method and doneness level. So we were unable to consider well-done meat or HAA intakes in our analyses. We also limited ourselves to arguably the single most important enzyme involved in the metabolism of HAAs. However, inter-individual variation in the activity of the other genes in this pathway would be more likely to dilute than to create spurious effects in the analyses conducted here.

In conclusion, this large study provides substantial support for a role of NAT2 in modifying the association between intake of red meat and, particularly, processed meat and colorectal cancer risk in Japanese and African Americans, two populations at high risk for this disease. Lowering consumption of red meat, especially processed meat, may be an effective approach for CRC prevention in these and other populations with a high frequency of the rapid NAT2 phenotype.

## Methods

### Subjects, genotypes and quality control

The Japanese samples included 2,217 CRC cases and 3,788 controls from the Multiethnic Cohort study (MEC), the Fukuoka Colorectal Cancer Study (Fukuoka), the Japan Public Health Center cohort study (JPHC) and the Nagano Colorectal Cancer Study (Nagano). The African American samples included 527 cases and 4,527 controls from the MEC, the University of North Carolina Rectal Cancer Study (UNC) and the Prostate, Lung, Colorectal and Ovarian Cancer Screening (PLCO) Trial. Details on study design, genotyping and quality control procedures can be found in previous publications [33,34]. Information on basic demographics and lifestyle factors was obtained from in-person interviews and/or self-administered structured questionnaires. All participating studies were approved by their respective Institutional Review Board (University of Hawaii, Japan National Cancer Center, University of Kyushu, University of North Carolina, US National Cancer Institute) and had participants sign a consent form. The present GWAS study was approved by the University of Hawaii Human Studies Program.

Briefly, Japanese subjects were genotyped with the Illumina 1M-Duo or the Illumina 660W-Quad array and African Americans on the Illumina 1M-Duo or the Illumina Omni 2.5M arrays. Samples were excluded for low call rates, gender mismatch or being an ethnicity outlier or a close (≥ 2nd degree) relative to another subject. In addition, studies or subjects missing age, gender or body mass index (BMI) were excluded. Genotyped SNPs were excluded based on call rates, concordance rates among duplicate pairs, deviation from Hardy-Weinberg equilibrium among controls, Mendelian errors in family trios or poor clustering quality.

Compared to our previous report searching for genetic susceptibility variants for CRC [33], we restricted the present analysis to studies that included both cases and controls, because including studies without controls would generate differential distributions of “environmental” factors (due to differences in questionnaires and measurement error) across studies and could bias the associations between “environmental” variables and disease.

### Predicted NAT2 activity

NAT2 acetylation phenotype was inferred from a 7-SNP genotyping panel: G191A (R64Q, rs1801279), C282T (rs1041983), T341C (I114T, rs1801280), C481T (rs1799929), G590A (R197Q, rs1799930), A803G (K268R, rs1208) and G857A (G286E, rs1799931), as recommended previously [10,11]. Except for G590A (R197Q, rs1799930) and G857A (G286E, rs1799931) in the Japanese data (both imputed with R^2^ = 1), the other SNPs were genotyped in both ethnic groups. Haplotype phasing was performed with BEAGLE [35], using the 1000 Genomes Project (phase 1, release 3) East Asians as reference panels for the Japanese data, and Europeans and Africans as reference panels for the African American data. Individuals with two, one and no “rapid” alleles (NAT2*4, NAT2*11A, NAT2*12A, B, C and NAT2*13) were assigned to the “rapid”, “intermediate” and “slow” NAT2 phenotype category, respectively. Rs1495741 was genotyped in both ethnic groups.

### Statistical Analysis

The Japanese and African American studies were analyzed separately. Logistic regression was used to test for the main effects of NAT2, intakes of processed meat, red meat without processed meat, and total red meat, and the interactions between NAT2 and the meat intake variables on CRC risk, adjusting for age, sex, study, BMI and the first 4 principal components of genetic ancestry to control for population stratification. NAT2 was modeled both as a linear term (rapid /intermediate/slow). Meat intakes were grouped into 4 categories based on study-specific quartiles defined from the distributions among cases to maximize power. Interaction between NAT2 activity (slow = 1; intermediate = 2; rapid = 3) and meat intake quartiles (from 1 to 4) was tested using a cross-product interaction term. The p-values from Wald test for interactions within each ethnic group were verified by likelihood ratio test (LRT). Stratified analyses by NAT2 phenotype were performed to interpret interaction effects. Results in the Japanese and African American studies were combined in meta-analyses using a fixed-effect model and I^2^ was calculated to assess between-ethnic group heterogeneity [36].

In sensitivity analyses, we examined heterogeneity of the main effects for the meat variables by study within population (Japanese or African Americans) with the LRT. In both ethnic groups, we checked whether the main effects of the meat variables were modified by the additional adjustment for other risk factors [pack-years of smoking (0, <20, ≥20), calcium and folate intakes (from foods only for the Japanese and from foods and dietary supplements for the African Americans, based on data availability) and dietary fiber intake], where dietary factors were categorized into study-specific tertiles. In the Japanese data, where sample size was larger, we also checked whether the interactions between NAT2 and meat intakes were modified by this additional adjustment. In Japanese, the main effects of red meats and the interaction between NAT2 and red meats were also examined by anatomical subsite (colon vs. rectum).

It was reported that, in Caucasians, rs1495741 alone predicted the NAT2 phenotype inferred from the 7-SNP panel with high accuracy. We compared the agreement of the NAT2 phenotypes inferred from rs1495741 alone with those inferred from the 7-SNP panel with 3×3 contingency table, separately in Japanese and African Americans.

All analyses were performed with *R* 3.0 (The Comprehensive R Archive Network http://www.r-project.org/). All tests were 2-sided and used a significance level of 0.05.

## Supporting Information

S1 Table Associations between NAT2 and colorectal cancer in Japanese, African Americans and when the two groups were combined. (DOCX)

S2 Table Study-specific cut-points for 4-category red meat intake. (DOCX)

S3 Table Association (odds ratios and 95% confidence interval) between meat intake and colorectal cancer, with adjustment for additional risk factors. (DOCX)

S4 Table Association of meat intake with colorectal cancer in Japanese, stratified by NAT2 phenotype, with adjustment for additional risk factors. (DOCX)

S5 Table Correlation between rs1495741 genotype and inferred 7 SNP-based NAT2 phenotype in the Japanese and African American studies. (DOCX)
