# Supplementary material for: Interaction between Red Meat Intake and NAT2 Genotype in Increasing the Risk of Colorectal Cancer in Japanese and African Americans
Source: PLoS One. 2015 Dec 18;10(12):e0144955. doi: 10.1371/journal.pone.0144955 (PMC4684304; doi:10.1371/journal.pone.0144955)
Supplement: S1 Table — (DOCX) [file pone.0144955.s001.docx]

**Supplementary Materials**

**Table S1**. Associations between NAT2 and colorectal cancer in Japanese, African Americans and when the two groups were combined.

|  |  |  | NAT2 | | |  |
| --- | --- | --- | --- | --- | --- | --- |
|  | Cases | Controls | Slow | Intermediate | Rapid | P_trend_ |
| Japanese | 2186 | 3736 | 1 | 1.06 (0.87, 1.28) | 1.05 (0.87, 1.27) | 0.77 |
| African Americans | 466 | 4356 | 1 | 0.91 (0.69, 1.19) | 0.75 (0.50, 1.14) | 0.19 |
| Combined | 2652 | 8092 | 1 | 1.00 (0.86, 1.18) | 0.99 (0.83, 1.18) | 0.81 |

Adjusted for age, sex, BMI (continuous), the first 4 principal components and sub-study sites.
