# Supplementary material for: Interaction between Red Meat Intake and NAT2 Genotype in Increasing the Risk of Colorectal Cancer in Japanese and African Americans
Source: PLoS One. 2015 Dec 18;10(12):e0144955. doi: 10.1371/journal.pone.0144955 (PMC4684304; doi:10.1371/journal.pone.0144955)
Supplement: S2 Table — (DOCX) [file pone.0144955.s002.docx]

**Supplementary Information**

**Table S2**. Study-specific cut-points for 4-category red meat intake

|  |  | Japanese | | | | African American | | |
| --- | --- | --- | --- | --- | --- | --- | --- | --- |
|  | Percentile | FUKUOKA | NAGANO | JPHC | MEC | MEC | PLCO | UNC |
| Processed meat | 25% | 2.1 | 2.0 | 0 | 7.0 | 6.3 | 9.2 | 8.9 |
|  | 50% | 5.3 | 4.3 | 1.4 | 14.2 | 13.5 | 18.7 | 17.9 |
|  | 75% | 10.9 | 8.8 | 4.7 | 24.1 | 28.5 | 31.7 | 32.2 |
|  |  |  |  |  |  |  |  |  |
| Red meat without processed meat | 25% | 23.2 | 17.3 | 5.7 | 18.1 | 16.3 | 20.6 | 20.1 |
|  | 50% | 36.0 | 34.3 | 12.0 | 31.8 | 27.8 | 34.4 | 43.1 |
|  | 75% | 55.8 | 63.3 | 20.9 | 48.9 | 48.5 | 64.2 | 70.4 |
|  |  |  |  |  |  |  |  |  |
| Total red meat | 25% | 27.4 | 22.8 | 8.1 | 27.7 | 27.0 | 36.6 | 29.4 |
|  | 50% | 43.6 | 41.3 | 15.9 | 46.6 | 44.1 | 53.6 | 67.5 |
|  | 75% | 66.5 | 71.5 | 25.7 | 74.0 | 76.1 | 102.5 | 108.0 |

Abbreviations: FUKUOKA, The Fukuoka Colorectal Cancer Study; NAGANO, the Nagano Colorectal Cancer Study; JPHC, Japan Public Health Center-based prospective study; MEC, Multiethnic Cohort; PLCO, Prostate, Lung, Colorectal, and Ovarian Cancer Screening Trial; UNC, The North Carolina Rectal Cancer Study.

The 4 categories (Q1 to Q4) for red meat variables were assigned sequentially for values in intervals ≤25, (25, 50], (50, 75], and >75 percentiles.
