# Supplementary material for: Interaction between Red Meat Intake and NAT2 Genotype in Increasing the Risk of Colorectal Cancer in Japanese and African Americans
Source: PLoS One. 2015 Dec 18;10(12):e0144955. doi: 10.1371/journal.pone.0144955 (PMC4684304; doi:10.1371/journal.pone.0144955)
Supplement: S3 Table — (DOCX) [file pone.0144955.s003.docx]

**Supplementary Information**

**Table S3. Association (odds ratios and 95% confidence interval) between meat intake and colorectal cancer, with adjustment for additional risk factors**

|  | Cases | Controls | Quartile 1 | Quartile 2 | Quartile 3 | Quartile 4 | P_trend_ | I^2^ (%) |
| --- | --- | --- | --- | --- | --- | --- | --- | --- |
| **Processed meat** | | |  |  |  |  |  |  |
| **Japanese** | 2161 | 3676 | 1.0 | 1.02 (0.87, 1.20) | 1.12 (0.96, 1.31) | 1.44 (1.21, 1.7) | 4.8e-5 |  |
| **African American** | 369 | 3998 | 1.0 | 1.16 (0.84, 1.59) | 1.09 (0.79, 1.49) | 1.20 (0.85, 1.69) | 0.39 |  |
| **Combined** | 2530 | 7674 | 1.0 | 1.05 (0.91, 1.21) | 1.11 (0.97, 1.28) | 1.39 (1.19, 1.61) | 5.7e-5 | 8 |
|  |  |  |  |  |  |  |  |  |
| **Red meat without processed meat** | | |  |  |  |  |  |  |
| **Japanese** | 2161 | 3676 | 1.0 | 1.14 (0.97, 1.34) | 1.18 (1, 1.39) | 1.34 (1.13, 1.58) | 0.0009 |  |
| **African American** | 369 | 3998 | 1.0 | 1.29 (0.94, 1.79) | 1.17 (0.85, 1.6) | 1.13 (0.81, 1.58) | 0.57 |  |
| **Combined** | 2530 | 7674 | 1.0 | 1.17 (1.01, 1.35) | 1.18 (1.02, 1.36) | 1.29 (1.11, 1.50) | 0.0013 | 3 |
|  |  |  |  |  |  |  |  |  |
| **Total Red meat** | |  |  |  |  |  |  |  |
| **Japanese** | 2161 | 3676 | 1.0 | 1.19 (1.01, 1.39) | 1.15 (0.98, 1.35) | 1.40 (1.19, 1.66) | 0.0004 |  |
| **African American** | 369 | 3998 | 1.0 | 1.65 (1.20, 2.28) | 1.33 (0.97, 1.84) | 1.33 (0.95, 1.87) | 0.16 |  |
| **Combined** | 2530 | 7674 | 1.0 | 1.27 (1.10, 1.46) | 1.18 (1.03, 1.36) | 1.39 (1.19, 1.62) | 0.0001 | 0 |

Adjusted for age, sex, BMI (continuous), the first 4 principal components and sub-study sites, folate and calcium intake from food, dietary fiber, and pack-years of smoking.

Sample sizes were reduced due to sporadic missing values in covariates.
