# Supplementary material for: Interaction between Red Meat Intake and NAT2 Genotype in Increasing the Risk of Colorectal Cancer in Japanese and African Americans
Source: PLoS One. 2015 Dec 18;10(12):e0144955. doi: 10.1371/journal.pone.0144955 (PMC4684304; doi:10.1371/journal.pone.0144955)
Supplement: S4 Table — (DOCX) [file pone.0144955.s004.docx]

**Supplementary Information**

**Table S4**. **Association of meat intake with colorectal cancer in Japanese, stratified by NAT2 phenotype, with adjustment for additional risk factors**

|  | NAT2 | Cases | Controls | Quartile 1 | Quartile 2 | Quartile 3 | Quartile 4 | P_trend_ |
| --- | --- | --- | --- | --- | --- | --- | --- | --- |
| Processed meat (p_interaction_ = 0.075) | Slow | 212 | 373 | 1 | 0.87 (0.53, 1.43) | 1.13 (0.69, 1.86) | 1.20 (0.70, 2.07) | 0.39 |
|  | Intermediate | 931 | 1580 | 1 | 0.91 (0.71, 1.16) | 1.00 (0.79, 1.27) | 1.35 (1.04, 1.75) | 0.04 |
|  | Rapid | 1018 | 1723 | 1 | 1.20 (0.95, 1.52) | 1.25 (0.99, 1.57) | 1.64 (1.28, 2.10) | 0.00020 |
|  |  |  |  |  |  |  |  |  |
| Red meat without processed meat (p_interaction_ = 0.23) | Slow | 212 | 373 | 1 | 1.05 (0.64, 1.72) | 1.19 (0.70, 2.02) | 1.15 (0.68, 1.95) | 0.53 |
|  | Intermediate | 931 | 1580 | 1 | 1.04 (0.81, 1.33) | 1.22 (0.95, 1.56) | 1.31 (1.01, 1.69) | 0.022 |
|  | Rapid | 1018 | 1723 | 1 | 1.28 (1.01, 1.61) | 1.17 (0.93, 1.49) | 1.45 (1.13, 1.86) | 0.010 |
|  |  |  |  |  |  |  |  |  |
| Total red meat (p_interaction_ = 0.089) | Slow | 212 | 373 | 1 | 1.66 (1.00, 2.73) | 1.20 (0.70, 2.05) | 1.28 (0.76, 2.14) | 0.59 |
|  | Intermediate | 931 | 1580 | 1 | 1.13 (0.88, 1.44) | 1.11 (0.87, 1.42) | 1.36 (1.05, 1.77) | 0.04 |
|  | Rapid | 1018 | 1723 | 1 | 1.17 (0.92, 1.48) | 1.19 (0.94, 1.50) | 1.52 (1.18, 1.94) | 0.0018 |

Adjusted for age, sex, BMI (continuous), the first 4 principal components and sub-study sites, folate and calcium intake from food, dietary fiber, and pack-years of smoking.

Sample sizes were reduced due to sporadic missing values in covariates.
