# Supplementary material for: Interaction between Red Meat Intake and NAT2 Genotype in Increasing the Risk of Colorectal Cancer in Japanese and African Americans
Source: PLoS One. 2015 Dec 18;10(12):e0144955. doi: 10.1371/journal.pone.0144955 (PMC4684304; doi:10.1371/journal.pone.0144955)
Supplement: S5 Table — (DOCX) [file pone.0144955.s005.docx]

**Supplementary Information**

**Table S5**. Correlation between rs1495741 genotype and inferred 7 SNP-based NAT2 phenotype in the Japanese and African American studies

|  |  |  | NAT2 | |  |  |  |  |
| --- | --- | --- | --- | --- | --- | --- | --- | --- |
|  | rs1495741 | Slow | Intermediate | Rapid | Total | %Agreement | Sensitivity | Specificity |
| Japanese | AA | 583 | 59 | 2 | 644 |  |  |  |
|  | AG | 19 | 2493 | 135 | 2647 | 95.8% | 96.8% | 98.9% |
|  | GG | 0 | 39 | 2675 | 2714 |  |  |  |
|  | Total | 602 | 2591 | 2812 | 6005 |  |  |  |
|  |  |  |  |  |  |  |  |  |
|  |  |  | NAT2 |  |  |  |  |  |
|  | rs1495741 | Slow | Intermediate | Rapid |  |  |  |  |
| African American | AA | 767 | 1247 | 9 | 2023 |  |  |  |
|  | GA | 23 | 2226 | 92 | 2341 | 67.7% | 97.1% | 70.5% |
|  | GG | 0 | 260 | 430 | 690 |  |  |  |
|  | Total | 790 | 3733 | 531 | 5054 |  |  |  |

%Agreement is the proportion that the AA, AG and GG genotype respectively matched the slow, intermediate and rapid NAT phenotype

Sensitivity is the proportion of AA genotype among subjects with the NAT2 slow phenotype.

Specificity is the proportion of GG and AG genotypes among subjects with the NAT2 rapid/intermediate phenotype
